# PlexinD1 signaling controls domain-specific dendritic development in newborn neurons in the postnatal olfactory bulb

**DOI:** 10.3389/fnins.2023.1143130

**Published:** 2023-07-13

**Authors:** Masato Sawada, Ayato Hamaguchi, Naomichi Mano, Yutaka Yoshida, Akiyoshi Uemura, Kazunobu Sawamoto

**Affiliations:** ^1^Department of Developmental and Regenerative Neurobiology, Institute of Brain Science, Nagoya City University Graduate School of Medical Sciences, Nagoya, Japan; ^2^Division of Neural Development and Regeneration, National Institute of Physiological Sciences, Okazaki, Japan; ^3^Burke Neurological Institute, White Plains, NY, United States; ^4^Brain and Mind Research Institute, Weill Cornell Medicine, New York, NY, United States; ^5^Neural Circuit Unit, Okinawa Institute of Science and Technology Graduate University, Okinawa, Japan; ^6^Department of Retinal Vascular Biology, Nagoya City University Graduate School of Medical Sciences, Nagoya, Japan

**Keywords:** postnatal neurogenesis, ventricular-subventricular zone, olfactory bulb, newborn neurons, dendrites, PlexinD1, RhoJ

## Abstract

Newborn neurons show immature bipolar morphology and continue to migrate toward their destinations. After the termination of migration, newborn neurons undergo spatially controlled dendrite formation and change into a complex morphology. The mechanisms of dendritic development of newborn neurons have not been fully understood. Here, we show that in the postnatal olfactory bulb (OB), the Sema3E-PlexinD1 signaling, which maintains bipolar morphology of newborn neurons, also regulates their dendritic development after the termination of migration in a dendritic domain-specific manner. Genetic ablation of *Sema3E* or *PlexinD1* enhanced dendritic branching in the proximal domain of the apical dendrites of OB newborn granule cells, whereas PlexinD1 overexpression suppressed it in a Rho binding domain (RBD)-dependent manner. Furthermore, RhoJ, a small GTPase that directly binds to PlexinD1RBD in vascular endothelial cells, is expressed in migrating and differentiating newborn granule cells in the OB and is also involved in the suppression of proximal branching of their apical dendrites. These results suggest that the Sema3E-PlexinD1-RhoJ axis regulates domain-specific dendrite formation of newborn neurons in the postnatal OB.

## Introduction

The brain of postnatal mammals contains neural stem cells (NSCs), which have the ability to generate new functional neurons. In the ventricular-subventricular-zone (V-SVZ) lining the lateral walls of the lateral ventricles, the largest neurogenic niche in the postnatal brain, newborn neurons generated from NSCs migrate toward the olfactory bulb (OB), a primary center for odor information processing (Bressan and Saghatelyan, 2020; Nakajima et al., 2021). After reaching the OB, most of the newborn neurons terminate their migration in the granule cell layer (GCL), develop their complex dendrites to precisely connect with preexisting OB circuits, and differentiate fully into granule cells, the major inhibitory interneurons in the OB (Luskin, 1993; Lois and Alvarez-Buylla, 1994). Eliminating newborn neurons or blocking their synaptic transmission causes disruption of normal olfactory behaviors (Breton-Provencher et al., 2009; Sakamoto et al., 2014; Muthusamy et al., 2017). Thus, the integration of newborn neurons into mature OB circuits is critical for olfactory functions, and could be accomplished by appropriate dendritogenesis. However, its mechanism is still largely unknown.

In the OB, granule cells have short basal dendrites extending in the basal domain and a single long apical dendrite consisting of an unbranched proximal domain and a highly ramified distal domain (Price and Powell, 1970; Petreanu and Alvarez-Buylla, 2002; Kelsch et al., 2008). In the apical dendrite of granule cells, while the proximal domain receives centrifugal inputs from olfactory higher centers, the distal domain receives peripheral inputs from mitral/tufted cells, projection neurons in the OB (Shepherd et al., 2004). Therefore, the dendritic domains of granule cells are integrated into functionally distinct OB circuits, and their morphological regulation could be the basis of granule cell functions. Previous studies suggest that olfactory input (Saghatelyan et al., 2005) and 5 T4 (Yoshihara et al., 2012) promote dendritic branching in the distal domain of the apical dendrites. However, the mechanism for suppression of dendritic branching in the proximal domain of apical dendrites remains unknown.

The secreted protein Sema3E and its receptor PlexinD1 are involved in morphogenesis of the nervous and vascular systems during development (Gay et al., 2011; Oh and Gu, 2013). In the central nervous system (CNS), PlexinD1 signaling is involved in the regulation of migration (Bribian et al., 2014; Sawada et al., 2018), survival (Cariboni et al., 2015), axonal elongation (Chauvet et al., 2007; Deck et al., 2013; Burk et al., 2017), and connections (Pecho-Vrieseling et al., 2009; Ding et al., 2011; Fukuhara et al., 2013; Mata et al., 2018) of neural cells. However, the role of PlexinD1 signaling in dendritogenesis in the CNS remains unclear. We have previously shown that PlexinD1 signaling suppresses the formation of filopodium-like lateral protrusions (FLPs), which branch laterally from the proximal domain of the leading process of migrating newborn neurons, thereby maintaining their immature bipolar morphology (Sawada et al., 2018). Therefore, we hypothesized that PlexinD1 signaling also suppresses dendritic branching in the proximal domain of apical dendrites in newborn granule cells after migration termination.

In this study, we show that PlexinD1 signaling is involved in the suppression of proximal branching of apical dendrites in newborn granule cells. We also show that this domain-specific regulation is controlled by the small GTPase RhoJ, a direct binding partner of PlexinD1.

## Methods

### Animals

Wild-type (WT) C57BL/6 J mice were purchased from Japan SLC. *PlexinD1*-flox mice (Zhang et al., 2009) were described previously. *Sema3E-*KO mice (Gu et al., 2005) were provided by Dr. Fanny Mann (Institut de Biologie du Developpement de Marseille) and Dr. Christopher E. Henderson (Columbia University). *RhoJ*-KO mice were described previously (Kim et al., 2014). Both male and female animals were used in this study. All animals were maintained with their mother mouse during weaning and within 7 mice per cage after weaning on a 12 h light/dark cycle with *ad libitum* access to food and water. All of the animal experiments were performed in accordance with the guidelines and regulations of Nagoya City University (Approval No. 21-028).

### Lentiviral vectors and plasmids

CSII-CMV-RfA-IRES2-Venus and CSII-EF-Venus lentiviral vectors were provided by Dr. Hiroyuki Miyoshi (RIKEN Tsukuba BioResource Center). CSII-CMV-PlexinD1-IRES2-Venus and CSII-CMV-PlexinD1ΔRBD-IRES2-Venus were described previously (Sawada et al., 2018). The pLV-CMV-tdTomato-IRES-Cre was provided by Dr. Magdalena Götz (Munich University). These viral vectors and the packaging vectors (pCAG-HIVgp and pCMV-VSV-G-RSV-Rev) were co-transfected into HEK293T cells using polyethylenimine to generate lentiviral particles, and then the culture supernatants were concentrated by centrifuging at 8,000 rpm for 16 h at 4°C using a refrigerated microcentrifuge (MX-307, Tomy) and resuspended with sterile phosphate buffered saline (PBS).

pEGFPC2 and pEGFPC2-RhoJ were described previously (Kusuhara et al., 2012; Fukushima et al., 2020). pCAGGS-DsRed was described previously (Sawada et al., 2018). These plasmids were amplified using *E. coli* and purified using a PureLink HiPure Plasmid Midiprep kit (Invitrogen).

### *In vitro* V-SVZ cell culture

The V-SVZ cell culture was performed as described previously (Sawada et al., 2018). Briefly, the V-SVZ tissues were dissected from P0-1 WT, *PlexinD1^+/fl^*, and *PlexinD1^fl/fl^* pups and dissociated with trypsin–EDTA (Invitrogen). The cells were washed two times in L-15 medium (Invitrogen) containing 40 μg/mL DNase I (Roche), seeded on coverglass (Matsunami) in 24-well cell culture plates, and cultured in Neurobasal medium (GIBCO) containing 10% fetal bovine serum, 2% NeuroBrew-21 (MACS Miltenyi Biotec), 2 mM L-glutamine (GIBCO), and 50 U/mL penicillin–streptomycin (GIBCO). At 4 days *in vitro* (div), 1 μL of lentiviral particles was added into the culture medium. At 10 div, cells were fixed with 4% paraformaldehyde (PFA) in 0.1 M phosphate buffer (PB) for 15 min at room temperature (RT) and subjected to immunocytochemistry.

### *In vivo* lentiviral infection and electroporation

Injection of lentiviral suspension and plasmid solution was described previously (Ota et al., 2014; Sawada et al., 2018). For lentiviral infection, a 2 μL volume of lentiviral suspension was stereotaxically injected into the V-SVZ (1.8 mm anterior, 1.4 mm lateral to lambda and 1.5–2.0 mm deep) of male and female pups at postnatal day 1 (P1). For *in vivo* electroporation of P1 male and female pups, a 2 μL volume of plasmids (3.5 μg/μL) containing 0.05% Fast Green was stereotaxically injected into the lateral ventricle (1.8 mm anterior, 1.2 mm lateral to lambda and 2.0 mm deep), and electroporation was performed using an electroporator (CUY-21SC, Nepagene) with a forceps-type electrode (CUY650P7, Nepagene). Lentivirus-injected or electroporated pups were allowed to recover on a heating pad and returned to their home cage.

### Immunohistochemistry and immunocytochemistry

Immunohistochemistry was performed as described previously (Sawada et al., 2018). Briefly, the brain was fixed by transcardiac perfusion with 4% PFA in 0.1 M PB, and postfixed overnight in the same fixative at 4°C. Sixty-micrometer-thick coronal sections were prepared using a vibratome (VT-1200S, Leica) and incubated for 30 min at RT in blocking solution (10% normal donkey serum and 0.2% Triton X-100 in PBS). The sections were then incubated overnight at 4°C with the primary antibodies, and then for 2 h at RT with biotin- or Alexa Fluor-conjugated secondary antibodies (1:1,000, Invitrogen) in the blocking solution. For immunocytochemistry, fixed cells were incubated for 30 min at RT in blocking solution, overnight at 4°C with the primary antibodies, and then for 2 h at RT with Alexa Fluor-conjugated secondary antibodies (1:1,000, Invitrogen) in the blocking solution. The signal amplification and visualization were performed using the Vectastain Elite ABC kit (Vector Laboratories) and Tyramide Signal Amplification (Thermo Fisher Scientific), respectively. The following primary antibodies were used: mouse anti-βIII-tubulin (Tuj1) (1:1,000, Sigma); rat anti-CD31 (1:100, BD Pharmingen); rabbit anti-doublecortin (Dcx) (1:200, Cell Signaling Technology); guinea pig anti-Dcx (1:500, Chemicon); rabbit anti-DsRed (1:1,000, Clontech); rabbit anti-GFP (1:500, MBL); rat anti-GFP (1:500, Nakalai Tesque); rabbit anti-NeuN (1:1,000, abcam); goat anti-PlexinD1 (1:100, abcam); goat anti-PlexinD1 (1:100, R&D systems) antibodies. Nuclei were stained with Hoechst 33342 (1:5,000, Sigma).

### Confocal image acquisition, quantification, and dendritic tracing of granule cells

Image acquisition and dendritic tracing of granule cells were performed as previously described (Sawada et al., 2018). Images of labeled granule cells of the postnatal OB were acquired by scanning at 2 μm intervals using an LSM 700 confocal laser-scanning microscope (Carl Zeiss) with a 20× objective lens (NA 0.8). For characterization of *RhoJ^+/GFP^*-positive cells, three regions-of-interest (ROIs) were randomly selected from three consecutive sections (one ROI per section) from every sixth 60 μm-thick OB section and image acquisition was performed, and all of the positive cells in the ROIs were counted. The obtained proportions of *RhoJ^+/GFP^*-positive cells were reported as mean ± SEM (*n* = 3 mice). Acquired z-stack images of virally labeled neurons were traced, reconstructed, and quantified using Neurolucida and Neurolucida Explorer software (MBF Bioscience) (Sawada et al., 2018). For analysis of apical and basal dendrites, all of the labeled granule cells showing complete neuronal morphologies observed in every sixth section were traced and analyzed. For analysis of dendritic branching in the proximal domain of the apical dendrite, all of the labeled granule cells showing >100 μm-length apical dendrites from soma were analyzed in this study. Dendritic branching in all domains of granule cells was expressed as branching in the dendritic domain per cell. For validation of PlexinD1 expression in neuronal culture, the signal intensity in the cell surface of labeled cells was measured using ZEN software (Carl Zeiss), and the average signal intensity (per μm), and normalized value (average value of control groups is 1.0) were calculated as reported previously (Sawada et al., 2018).

### Statistical analysis

All of the data were two-tailed and analyzed using EZR (Kanda, 2013). The data distribution was analyzed by the Kolmogorov–Smirnov test. The equality of variance between groups was analyzed by the *F* test. Comparisons between two groups were analyzed by unpaired *t*-test or Mann–Whitney *U*-test. Comparisons among multiple groups were analyzed by one-way ANOVA test followed by a post-hoc Tukey–Kramer test, or Kruskal-Wallis test followed by a *post-hoc* Steel-Dwass test. All the numerical data are presented as the mean ± SEM and *p*-values less than 0.05 were considered statistically significant.

## Results

### PlexinD1 signaling specifically suppresses dendritic branching in the proximal domain of the apical dendrite of newborn granule cells in the postnatal OB

In the GCL of the postnatal OB, while Sema3E is expressed in mature granule cells, PlexinD1 is expressed in the leading process of migrating newborn neurons and is involved in suppressing FLP formation to maintain their immature morphology (Sawada et al., 2018). In this study, we investigated whether Sema3E-PlexinD1 signaling is also involved in the suppression of dendritic branching in the proximal domain of the apical dendrite of newborn granule cells that have terminated their migration.

**Figure 1:**
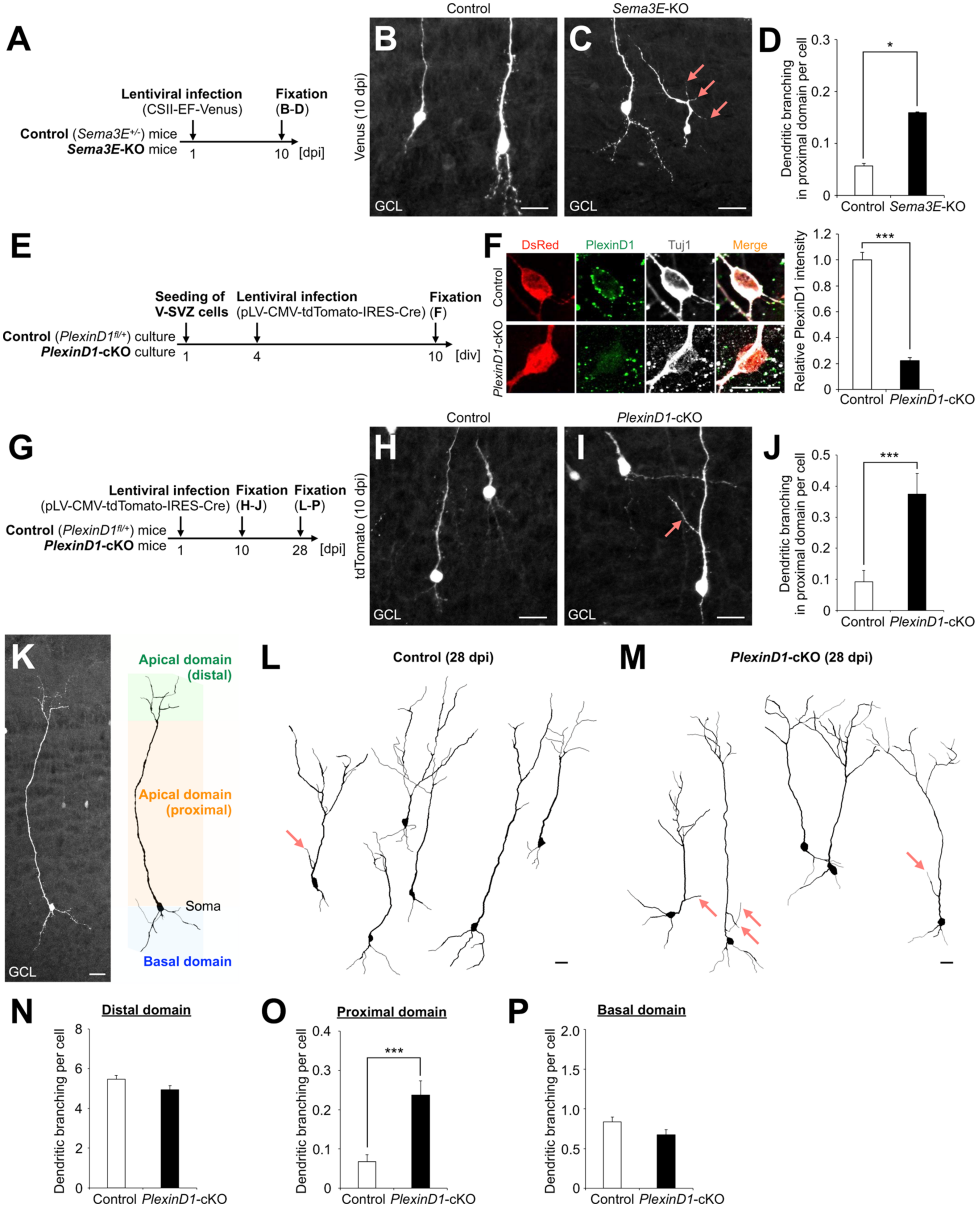
Sema3E-PlexinD1 signaling suppresses dendritic branching in the proximal domain of the apical dendrite of granule cells in the postnatal OB. **(A)** Experimental scheme in *Sema3E*-KO mice. **(B,C)** Representative projection images of Venus+ granule cells in control **(B)** and *Sema3E*-KO **(C)** mice at 10 dpi. **(D)** Dendritic branch number in the proximal domain of the apical dendrite in control (*n* = 527 cells from 4 mice) and *Sema3E*-KO (*n* = 962 cells from 5 mice) mice. **(E)** Experimental scheme of *PlexinD1*-cKO culture. **(F)** Representative images of DsRed+ (red) Tuj1+ (white) cultured control and *PlexinD1*-cKO neurons. Green indicates PlexinD1. Graph indicates relative PlexinD1 intensity in the infected neurons (control, *n* = 35 cells; *PlexinD1*-cKO, *n* = 30 cells; three independent experiments). **(G)** Experimental scheme in *PlexinD1*-cKO mice. **(H,I)** Representative projection images of Venus+ granule cells in Control **(H)** and *PlexinD1*-cKO **(I)** mice at 10 dpi. **(J)** Dendritic branch number in the proximal domain of the apical dendrite in control (*n* = 206 cells from 6 mice) and *PlexinD1*-cKO (*n* = 139 cells from 5 mice) mice at 10 dpi. **(K)** Classification of dendritic domains in granule cells. **(L,M)** Representative dendritic tracing of Venus+ granule cells in control [**(L)**; *n* = 200 cells from 5 mice] and *PlexinD1*-cKO [**(M)**; *n* = 138 cells from 5 mice] mice. **(N–P)** Dendritic branch number in the distal [**(N)**; control, *n* = 200 cells from 5 mice; KO, *n* = 138 cells from 5 mice], proximal [**(O)**; control, *n* = 200 cells from 5 mice; KO, *n* = 138 cells from 5 mice], and basal [**(P)**; control, *n* = 200 cells from 5 mice; KO, *n* = 138 cells from 5 mice] domains in control and *PlexinD1*-cKO mice at 28 dpi. Pink arrows indicate dendritic branches in the proximal domain of apical dendrites. GCL, granule cell layer. *p < 0.05, ***p < 0.005. Scale bars: **(B)**, **(C)**, **(H)**, **(I)**, **(K)**, 20  μm; **(F)**, 10 μm. Bars indicate mean ± SEM.

First, we injected lentivirus encoding Venus into the V-SVZ of *Sema3E^+/−^* (control) and *Sema3E^−/−^* (*Sema3E*-KO) mice (Gu et al., 2005) at postnatal day 1 (P1) and analyzed the dendritic morphology of Venus+ granule cells in the GCL at 10 days post infection (dpi) (Figure 1A). The branch number in the proximal domain of the apical dendrite was significantly increased in *Sema3E*-KO mice compared with control mice (Figures 1B–D), suggesting that Sema3E suppresses dendritic branching in the proximal domain of the apical dendrite of newborn granule cells.

Next, to investigate the expression and role of PlexinD1 in this process, brain sections were stained with a PlexinD1 antibody. The PlexinD1 signal was observed in the proximal domain of the apical dendrite of differentiating granule cells (Supplementary Figure S1A). For functional analyses of PlexinD1, we used lentivirus encoding tdTomato and Cre recombinase. The decrease of PlexinD1 proteins by these vectors was confirmed in primary neuronal cultures (Figures 1E,F). To examine the function of PlexinD1 *in vivo*, we injected these lentiviral vectors into the V-SVZ of *PlexinD1^+/flox^* (control) and *PlexinD1^flox/flox^* (*PlexinD1*-cKO) mice (Zhang et al., 2009) at P1 and analyzed the dendritic morphology of tdTomato+ granule cells (Figure 1G). At 10 dpi, similar to the phenotype observed in *Sema3E*-KO mice, the branch number in the proximal domain of the apical dendrite was significantly increased by *PlexinD1* deficiency (Figures 1H–J). Moreover, at 28 dpi, when granule cells morphologically mature (Petreanu and Alvarez-Buylla, 2002), the whole dendritic morphology of tdTomato+ granule cells was traced (Figure 1K). We found that the dendritic branch number in the proximal but not the distal or basal domain in tdTomato+ granule cells was significantly increased in *PlexinD1*-cKO mice compared to that in control mice (Figures 1L–P). The dendritic length of the apical and basal dendrites was not significantly different between control and *PlexinD1*-cKO mice (Supplementary Figures S1B–E). Taken together, these results suggest that Sema3E-PlexinD1 signaling is specifically involved in the suppression of dendritic branching in the proximal domain of the apical dendrite of newborn granule cells in the postnatal OB.

### PlexinD1’s RBD is involved in the PlexinD1-mediated suppression of dendritic branching at proximal domain of apical dendrites in newborn granule cells

**Figure 2:**
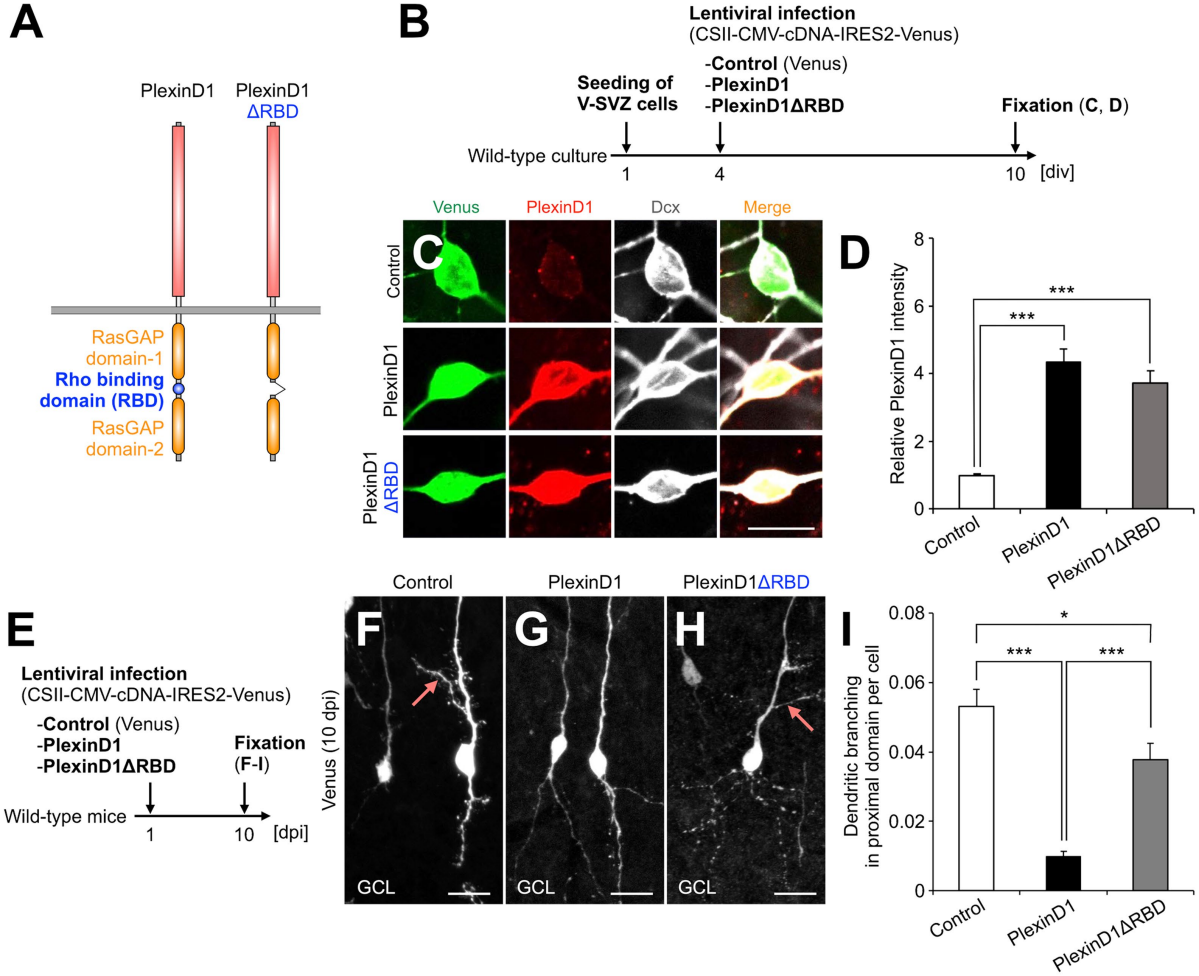
PlexinD1’s RBD is involved in the PlexinD1-mediated suppression of lateral dendrite formation in granule cells in the postnatal OB. **(A)** Molecular structure of PlexinD1. **(B)** Experimental scheme of PlexinD1- and PlexinD1ΔRBD-overexpressing neuronal culture. **(C)** Representative images of Venus+ (green) Dcx + (white) cultured control, PlexinD1-overexpressing, and PlexinD1ΔRBD-overexpressing neurons. Red indicates PlexinD1. **(D)** Relative PlexinD1 intensity in the infected neurons (control, *n* = 49 cells; PlexinD1, *n* = 38 cells; PlexinD1ΔRBD, *n* = 30 cells; three independent experiments). **(E)** Experimental scheme for PlexinD1 overexpression *in vivo*. **(F–H)** Representative projection images of Venus+ control **(F)**, PlexinD1-overexpressing **(G)**, and PlexinD1ΔRBD-overexpressing **(H)** granule cells at 10 dpi. **(I)** Proportions of lateral dendrite-bearing granule cells at 10 dpi (control, *n* = 2,217 cells from 5 mice; PlexinD1, *n* = 4,204 cells from 5 mice; PlexinD1ΔRBD, *n* = 1,641 cells from 5 mice). Pink arrows indicate dendritic branches in the proximal domain of the apical dendrite. GCL, granule cell layer; RBD, Rho binding domain. *p < 0.05, ***p < 0.005. Scale bars: **(C)**, 10  μm; **(F–H)**, 20  μm. Bars indicate mean ± SEM.

The intracellular domain of Plexins has a Rho binding domain (RBD), which regulates cytoskeletal dynamics through binding to various Rho family small GTPases (Gay et al., 2011) (Figure 2A). To study the role of PlexinD1 and its RBD in dendritic development of newborn granule cells, we generated lentiviral vectors encoding PlexinD1 or its deletion mutant of RBD (PlexinD1ΔRBD), and confirmed their overexpression in primary neuronal cultures (Figures 2A–D). To examine the effects of PlexinD1 overexpression on the dendritic branching *in vivo*, we injected these lentiviruses into the V-SVZ and analyzed the dendritic morphology of infected granule cells in the OB at 10 dpi (Figure 2E). Overexpression of PlexinD1 decreased the number of dendritic branches in the proximal domain of the apical dendrite (Figures 2F,G,I). This effect was partially diminished when lentiviruses encoding PlexinD1ΔRBD were injected (Figures 2H,I). These results suggest that RBD is involved in the suppression of dendritic branching in the proximal domain of the apical dendrites of newborn granule cells by PlexinD1 overexpression.

### Rhoj is expressed in migrating and differentiating granule cells, and suppresses dendritic branching of proximal domain of their apical dendrites

RhoJ, a member of Rho family small GTPases, is highly expressed in vascular endothelial cells and involved in Sema3E’s repulsive effect by directly binding to the PlexinD1RBD to promote F-actin depolymerization (Fukushima et al., 2011, 2020). In the developing retina, RhoJ is expressed in not only vascular endothelial cells but also neurons (Fukushima et al., 2020). However, the function of RhoJ in the CNS remains unknown. Since RBD is involved in the PlexinD1-induced suppression of dendritic branching in the proximal domain of apical dendrites (Figure 2), we hypothesized that RhoJ contributes to this process.

**Figure 3:**
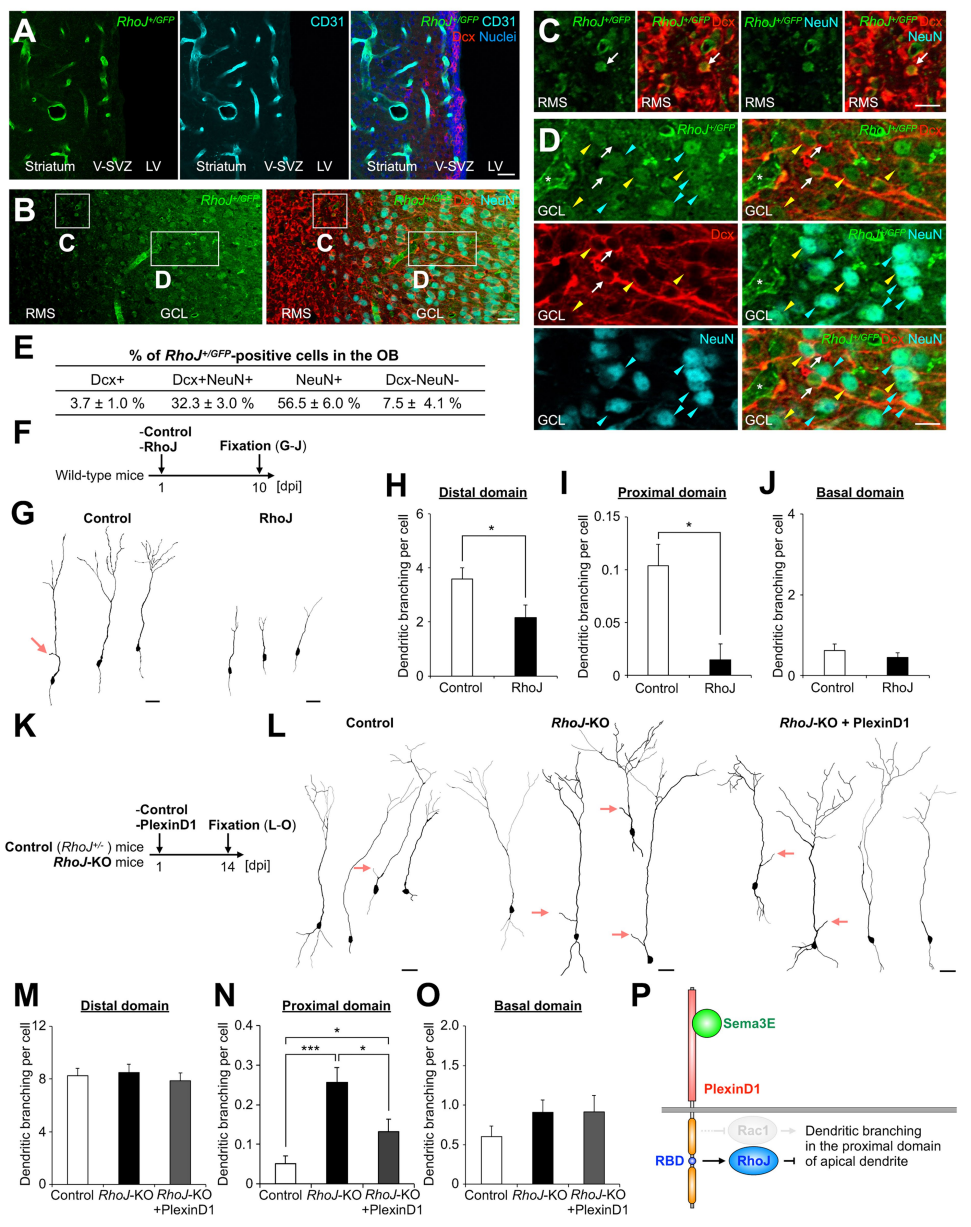
RhoJ is expressed in migrating and differentiating granule cells in the postnatal OB and involved in the suppression of their dendritic branching in the proximal domain of the apical dendrite. **(A)** Representative images of the coronal V-SVZ sections in *RhoJ^+/GFP^* mice stained for GFP (green), Dcx (red), and CD31 (cyan). Nuclei were stained with Hoechst 33342 (Blue). **(B–D)** Representative images of the coronal OB sections in *RhoJ^+/GFP^* mice stained for GFP (green), Dcx (red), and NeuN (cyan). Boxed area in **(B)** was enlarged in **(C)** and **(D)**. White arrows, yellow arrowheads, and cyan arrowheads **(C)** and **(D)** indicate GFP + Dcx + NeuN-, GFP + Dcx + NeuN+, and GFP + Dcx-NeuN+ granule cells, respectively. **(E)** Proportions of *RhoJ^+/GFP^*-positive cells in the OB (*n* = 3 mice; 144 cells analyzed). **(F)** Experimental scheme for RhoJ overexpression experiment. **(G)** Representative dendritic tracings of control (*n* = 32 cells from 4 mice) and RhoJ-overexpressing (*n* = 36 cells from 8 mice) granule cells at 10  day-post injection (dpi). **(H–J)** Dendritic branch numbers of distal [**(H)**; control, *n* = 32 cells from 4 mice; RhoJ, *n* = 36 cells from 8 mice], proximal [**(I)**; control, *n* = 231 cells from 4 mice; RhoJ, *n* = 67 cells from 8 mice], and basal [**(J)**; control, *n* = 32 cells from 4 mice; RhoJ, *n* = 36 cells from 8 mice] domains in control and RhoJ-overexpressing granule cells at 10 dpi. **(K)** Experimental scheme for RhoJ loss-of-function experiment. **(L)** Representative dendritic tracings of control (*n* = 43 cells from 3 mice), *RhoJ*-KO (*n* = 47 cells from 3 mice), and PlexinD1-overexpressing *RhoJ*-KO (*n* = 24 cells from 4 mice) granule cells at 14 dpi. **(M–O)** Dendritic branch numbers of distal [**(M)**; control, *n* = 43 cells from 3 mice; *RhoJ*-KO, *n* = 47 cells from 3 mice; *RhoJ*-KO + PlexinD1, *n* = 24 cells from 4 mice], proximal [**(N)**; control, *n* = 137 cells from 3 mice; *RhoJ*-KO, *n* = 140 cells from 3 mice; *RhoJ*-KO + PlexinD1, *n* = 128 cells from 4 mice], and basal [**(O)**; control, *n* = 43 cells from 3 mice; *RhoJ*-KO, *n* = 47 cells from 3 mice; *RhoJ*-KO + PlexinD1, *n* = 24 cells from 4 mice] domains in control, *RhoJ*-KO, and PlexinD1-overexpressing *RhoJ*-KO granule cells at 14 dpi. **(P)** Mechanism of dendritic branching in the proximal domain of the apical dendrite in granule cells in the postnatal OB. Pink arrows indicate dendritic branches in the proximal domain of the apical dendrite. V-SVZ, ventricular-subventricular zone; LV, lateral ventricle; RMS, rostral migratory stream; GCL, granule cell layer. *p < 0.05, ***p < 0.005. Scale bars: **(A)**, **(B)**, **(G)**, **(L)**, 20 μm; **(C)**, **(D)**, 10 μm. Bars indicate mean ± SEM.

First, to identify RhoJ-expressing cells in the V-SVZ-OB pathway, we analyzed GFP-expressing cell types in *RhoJ^+/GFP^* mice (Kim et al., 2014), in which EGFP replaces exon 1 of the *RhoJ* gene and is expressed under the control of the endogenous *RhoJ* promoter. EGFP was strongly expressed in the CD31+ vascular endothelial cells not only in the retina (Fukushima et al., 2020) but also in the brain (Figure 3A). We found that EGFP was not expressed in Dcx + cells in the V-SVZ, but was expressed in a subset of Dcx + and NeuN+ cells in the core and GCL of the OB (Figures 3B–E). These results suggest that newborn neurons express RhoJ during migration and maturation in the postnatal OB.

Next, to investigate the effect of forced expression of RhoJ on the dendritogenesis of newborn granule cells, plasmids encoding an EGFP-fused RhoJ were introduced into the V-SVZ by *in vivo* electroporation, and the dendritic morphology of labeled granule cells in the GCL was analyzed at 10 dpi (Figure 3F). The forced expression of RhoJ in granule cells significantly suppressed both their dendritic branching in the distal and proximal domains (Figures 3G–J) and dendritic length in the distal domain (Figure 3G; control, 340.0 ± 28.5 μm; RhoJ, 224.5 ± 29.0 μm; *p* = 0.0063, unpaired *t*-test). These results suggest that RhoJ overexpression has an inhibitory effect on dendritic branching and outgrowth *in vivo*.

Finally, to study the function of RhoJ on dendritogenesis of newborn granule cells, we introduced plasmids encoding DsRed into the V-SVZ of *RhoJ^+/GFP^* (control) and *RhoJ^GFP/GFP^* (*RhoJ*-KO) mice by *in vivo* electroporation and analyzed their dendritic morphology (Figure 3K). The dendritic branch number in the proximal but not distal or basal domain in DsRed+ granule cells was significantly increased in *RhoJ*-KO mice (Figures 3L–O). The dendritic length of the apical and basal dendrites was not significantly different between control and *RhoJ*-KO mice (Supplementary Figures S1B,F–H). Furthermore, the increase of dendritic branching in the proximal domain in *RhoJ*-KO neurons was partially diminished by PlexinD1 overexpression (Figures 3L–O). Together, these results suggest that RhoJ is specifically involved in the suppression of dendritic branching in the proximal domain of the apical dendrite of newborn granule cells in the postnatal OB.

## Discussion

In this study, we demonstrated that Sema3E-PlexinD1 signaling is involved in the domain-specific dendritic branching of newborn granule cells in the postnatal OB. Furthermore, we showed that RhoJ, a small GTPase that directly binds to PlexinD1, is expressed in newborn granule cells during migration and maturation in the postnatal OB and involved in the proximal domain-specific inhibition of dendritic branching. These results provide new insights into the dendritogenesis of newborn neurons in the postnatal brain.

In the postnatal OB, granule cells receive centrifugal inputs in the proximal domain of the apical dendrite (Kaneko et al., 2006; Whitman and Greer, 2007; Yokoyama et al., 2011; Komano-Inoue et al., 2014). The distal domain of the apical dendrite undergoes many branchings, whereas the proximal domain maintains an unbranched state (Shepherd et al., 2004). Excessive centrifugal input to newborn granule cells may result in strong GABA-mediated feedback to mitral/tufted cells and excessively downregulated neuronal transmission from the OB toward the olfactory higher centers. Therefore, granule cells need a mechanism to inhibit dendritic branching in the proximal domain of the apical dendrite to prevent them from receiving too much centrifugal input. Our results indicate that PlexinD1 signaling not only determines the final positioning of newborn neurons by suppressing FLP formation during migration (Sawada et al., 2018), but also maintains the proximal domain of the apical dendrite unbranched after migration termination, thereby ensuring proper reception of centrifugal inputs.

Previous studies suggested that olfactory input (Saghatelyan et al., 2005) and 5 T4 (Yoshihara et al., 2012) promote dendritic branching in the distal domain of granule cells. In contrast, the mechanism inhibiting their dendritic branching in the proximal domain has remained unknown. In this study, *PlexinD1*- or *RhoJ*-deficiency specifically enhanced dendritic branching in the proximal domain of the apical dendrite without affecting overall dendritic morphology or length in newborn granule cells. In newborn neurons migrating in the OB, PlexinD1 signaling suppresses FLP formation without affecting leading process formation (Sawada et al., 2018). Moreover, newborn neurons start to express RhoJ during their migration and maturation in the OB. Thus, our results suggest that Sema3E-PlexinD1-RhoJ signaling is involved in the proximal domain-specific suppression of branching both in the leading process during migration (Sawada et al., 2018) and in the apical dendrite after migration termination (this study).

FLPs are the cellular protrusions that link termination of migration and initiation of dendritogenesis in newborn neurons in the postnatal OB, and the timing of their formation is regulated by local PlexinD1 endocytosis during the process of migration termination (Sawada et al., 2018). Rac1 is locally activated in the proximal domain of the leading process to form FLPs (Sawada et al., 2018). On the other hand, this study showed that RhoJ suppresses dendritic branching in the proximal domain of apical dendrites. Since RhoJ, which is activated by Sema3E-PlexinD1 signaling, is localized in the perinuclear region and involved in F-actin depolymerization in vascular endothelial cells (Fukushima et al., 2020), it is possible that RhoJ is also localized in the proximal leading process and involved in the proximal domain-specific suppression of branching in migrating and maturing newborn neurons. Considering that PlexinD1 overexpression effects are partially diminished by RBD deletion (Figure 2I) or *RhoJ* deficiency (Figure 3N), our findings suggest that in newborn neurons, PlexinD1 activates RhoJ and inactivates Rac1 to suppress FLP formation during maintenance of migration, and its downregulation by local endocytosis relieves Rac1 inhibition and decreases RhoJ activation to promote FLP formation during termination of migration (Figure 3P). Thus, we propose that the PlexinD1-mediated regulation of the two opposing Rho GTPases has an advantage in the efficient transition from maintenance to termination of migration by controlling their timing of the branching of newborn neurons.

## Conclusion

In this study, we showed a domain-specific mechanism for dendritic development in newborn granule cells in the postnatal OB. These results contribute to a better understanding of the development and function of newborn neurons in the postnatal OB circuits.

## Data availability statement

The raw data supporting the conclusions of this article will be made available by the authors, without undue reservation.

## Ethics statement

The animal study was reviewed and approved by Nagoya City University.

## Author contributions

MS, AH, and NM performed experiments. MS, AH, NM, YY, AU, and KS analyzed the data. MS and KS wrote the manuscript. All authors contributed to the article and approved the submitted version.

## Funding

This work was supported by research grants from Japan Agency for Medical Research and Development (AMED) (23gm1210007 to KS), Japan Society for the Promotion of Science (JSPS) KAKENHI (19H04757, 19H04785, 18KK0213, and 20H05700 to KS), (18 K14823 and 21 K06395 to MS), Bilateral Open Partnership Joint Research Projects (to KS), Core-to-core program “Neurogenesis Research & Innovation Center (NeuRIC)” (JPJSCCA20230007 to KS), Grant-in-Aid for Research at Nagoya City University (to MS and KS), and the Takeda Science Foundation (to MS and KS).

## Conflict of interest

The authors declare that the research was conducted in the absence of any commercial or financial relationships that could be construed as a potential conflict of interest.

## Publisher’s note

All claims expressed in this article are solely those of the authors and do not necessarily represent those of their affiliated organizations, or those of the publisher, the editors and the reviewers. Any product that may be evaluated in this article, or claim that may be made by its manufacturer, is not guaranteed or endorsed by the publisher.

## Supplementary material

SUPPLEMENTARY FIGURE S1 Dendritic length of newborn granule cells in PlexinD1-cKO and RhoJ-KO mice **(A)** Representative image of differentiating granule cells stained for Dcx (green) and PlexinD1 (red). Arrows indicate the signal of PlexinD1 protein in the proximal domain of the apical dendrite. **(B)** Classification of dendrites in newborn granule cells. **(C–H)** Length of apical dendrites **(C, F)**, dendritic branch in the proximal domain of the apical dendrite **(D, G)**, and basal dendrites **(E, H)** of granule cells in PlexinD1-cKO **(C–E)** and RhoJ-KO **(F–H)** mice. Scale bar, 10 µm. Bars indicate mean ± SEM.
