# Corrigendum: PlexinD1 signaling controls domain-specific dendritic development in newborn neurons in the postnatal olfactory bulb

**DOI:** 10.3389/fnins.2023.1338853

**Published:** 2023-11-28

**Authors:** Masato Sawada, Ayato Hamaguchi, Naomichi Mano, Yutaka Yoshida, Akiyoshi Uemura, Kazunobu Sawamoto

**Affiliations:** ^1^Department of Developmental and Regenerative Neurobiology, Institute of Brain Science, Nagoya City University Graduate School of Medical Sciences, Nagoya, Japan; ^2^Division of Neural Development and Regeneration, National Institute of Physiological Sciences, Okazaki, Japan; ^3^Burke Neurological Institute, White Plains, NY, United States; ^4^Brain and Mind Research Institute, Weill Cornell Medicine, New York, NY, United States; ^5^Neural Circuit Unit, Okinawa Institute of Science and Technology Graduate University, Okinawa, Japan; ^6^Department of Retinal Vascular Biology, Nagoya City University Graduate School of Medical Sciences, Nagoya, Japan

**Keywords:** postnatal neurogenesis, ventricular-subventricular zone, olfactory bulb, newborn neurons, dendrites, PlexinD1, RhoJ

In the published article, there was an error in Figures 2 and 3 as published. In Figures 2E and 3F, “Wilt-type” is a typographical error of “Wild-type”. The corrected Figures 2 and 3 appear below.

**Figure 2:**
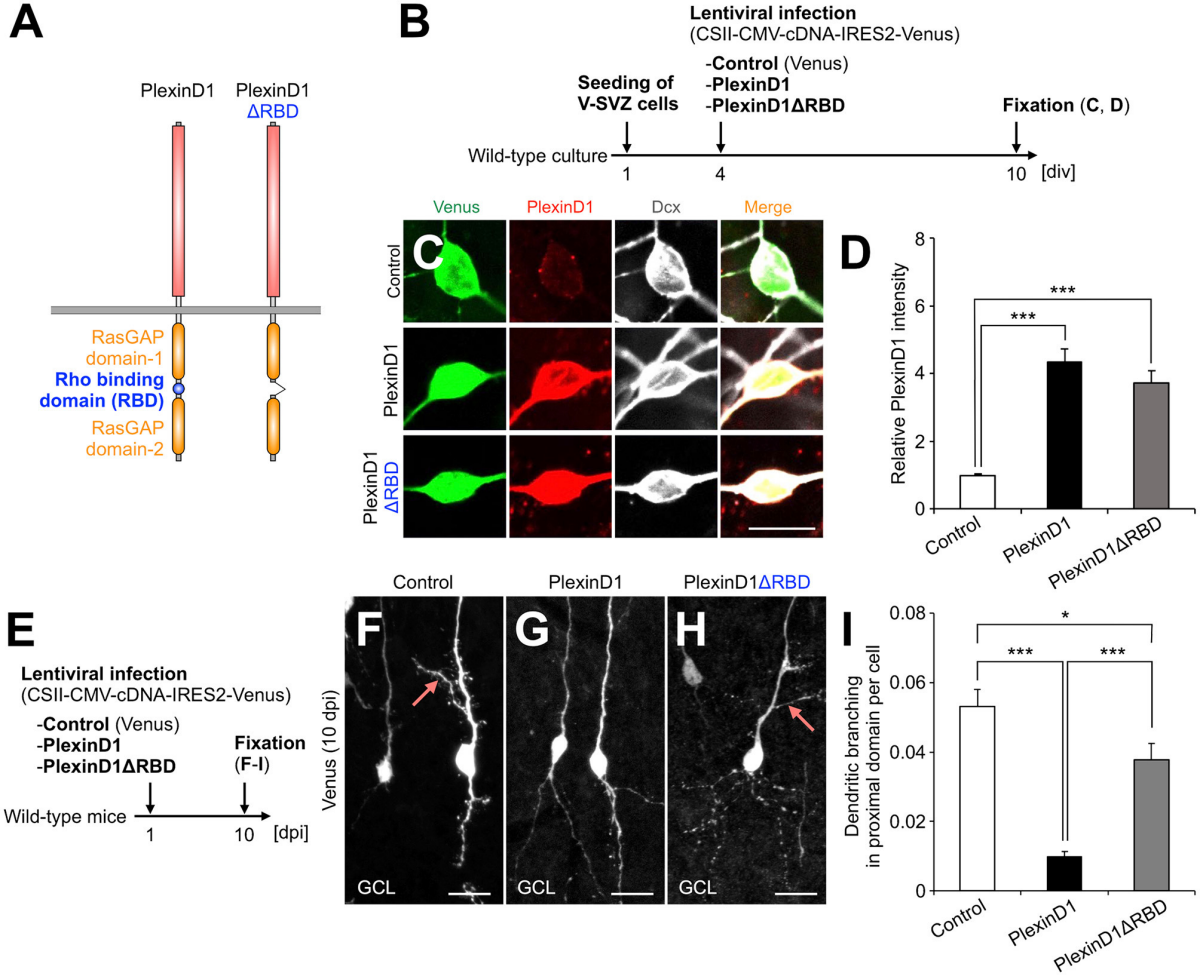
PlexinD1's RBD is involved in the PlexinD1-mediated suppression of lateral dendrite formation in granule cells in the postnatal OB. **(A)** Molecular structure of PlexinD1. **(B)** Experimental scheme of PlexinD1- and PlexinD1ΔRBD-overexpressing neuronal culture. **(C)** Representative images of Venus+ (green) Dcx+ (white) cultured control, PlexinD1-overexpressing, and PlexinD1ΔRBD-overexpressing neurons. Red indicates PlexinD1. **(D)** Relative PlexinD1 intensity in the infected neurons (control, *n* = 49 cells; PlexinD1, *n* = 38 cells; PlexinD1ΔRBD, *n* = 30 cells; three independent experiments). **(E)** Experimental scheme for PlexinD1 overexpression *in vivo*. **(F–H)** Representative projection images of Venus+ control **(F)**, PlexinD1-overexpressing **(G)**, and PlexinD1ΔRBD-overexpressing **(H)** granule cells at 10 dpi. **(I)** Proportions of lateral dendrite-bearing granule cells at 10 dpi (control, *n* = 2,217 cells from 5 mice; PlexinD1, *n* = 4,204 cells from 5 mice; PlexinD1ΔRBD, *n* = 1,641 cells from 5 mice). Pink arrows indicate dendritic branches in the proximal domain of the apical dendrite. GCL, granule cell layer; RBD, Rho binding domain. *p < 0.05, ***p < 0.005. Scale bars: **(C)**, 10 μm; **(F–H)**, 20 μm. Bars indicate mean ± SEM.

**Figure 3:**
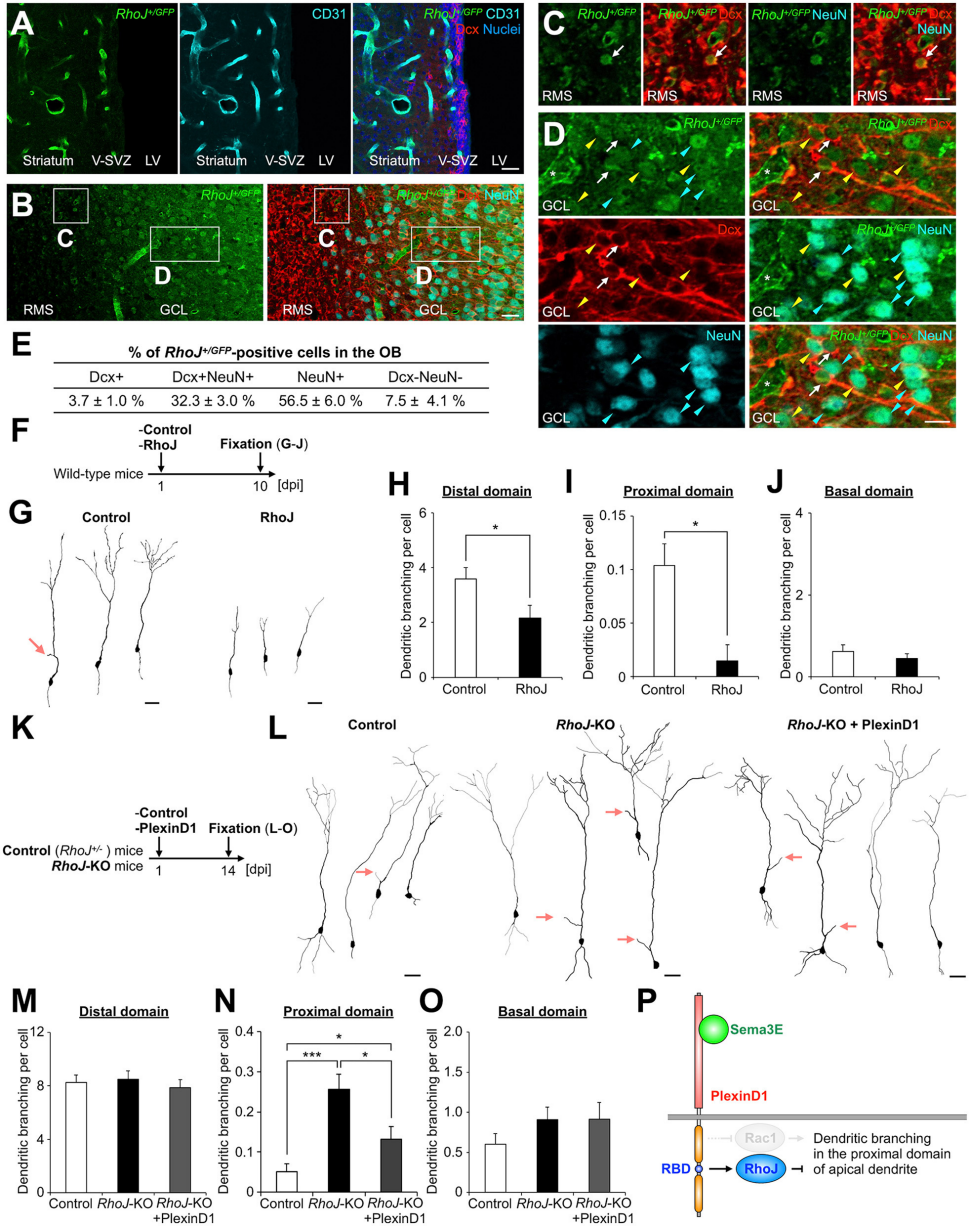
RhoJ is expressed in migrating and differentiating granule cells in the postnatal OB and involved in the suppression of their dendritic branching in the proximal domain of the apical dendrite. **(A)** Representative images of the coronal V-SVZ sections in *RhoJ*^+/*GFP*^ mice stained for GFP (green), Dcx (red), and CD31 (cyan). Nuclei were stained with Hoechst 33342 (Blue). **(B–D)** Representative images of the coronal OB sections in *RhoJ*^+/*GFP*^ mice stained for GFP (green), Dcx (red), and NeuN (cyan). Boxed area in **(B)** was enlarged in **(C)** and **(D)**. White arrows, yellow arrowheads, and cyan arrowheads **(C)** and **(D)** indicate GFP + Dcx + NeuN-, GFP + Dcx + NeuN+, and GFP + Dcx-NeuN+ granule cells, respectively. **(E)** Proportions of *RhoJ*^+/*GFP*^-positive cells in the OB (*n* = 3 mice; 144 cells analyzed). **(F)** Experimental scheme for RhoJ overexpression experiment. **(G)** Representative dendritic tracings of control (*n* = 32 cells from 4 mice) and RhoJ-overexpressing (*n* = 36 cells from 8 mice) granule cells at 10 day-post injection (dpi). **(H–J)** Dendritic branch numbers of distal [**(H)**; control, *n* = 32 cells from 4 mice; RhoJ, *n* = 36 cells from 8 mice], proximal [**(I)**; control, *n* = 231 cells from 4 mice; RhoJ, *n* = 67 cells from 8 mice], and basal [**(J)**; control, *n* = 32 cells from 4 mice; RhoJ, *n* = 36 cells from 8 mice] domains in control and RhoJ-overexpressing granule cells at 10 dpi. **(K)** Experimental scheme for RhoJ loss-of-function experiment. **(L)** Representative dendritic tracings of control (*n* = 43 cells from 3 mice), *RhoJ*-KO (*n* = 47 cells from 3 mice), and PlexinD1-overexpressing *RhoJ*-KO (*n* = 24 cells from 4 mice) granule cells at 14 dpi. **(M–O)** Dendritic branch numbers of distal [**(M)**; control, *n* = 43 cells from 3 mice; *RhoJ*-KO, *n* = 47 cells from 3 mice; *RhoJ*-KO + PlexinD1, *n* = 24 cells from 4 mice], proximal [**(N)**; control, *n* = 137 cells from 3 mice; *RhoJ*-KO, *n* = 140 cells from 3 mice; *RhoJ*-KO + PlexinD1, *n* = 128 cells from 4 mice], and basal [**(O)**; control, *n* = 43 cells from 3 mice; *RhoJ*-KO, *n* = 47 cells from 3 mice; *RhoJ*-KO + PlexinD1, *n* = 24 cells from 4 mice] domains in control, *RhoJ*-KO, and PlexinD1-overexpressing *RhoJ*-KO granule cells at 14 dpi. **(P)** Mechanism of dendritic branching in the proximal domain of the apical dendrite in granule cells in the postnatal OB. Pink arrows indicate dendritic branches in the proximal domain of the apical dendrite. V-SVZ, ventricular-subventricular zone; LV, lateral ventricle; RMS, rostral migratory stream; GCL, granule cell layer. *p < 0.05, ***p < 0.005. Scale bars: **(A)**, **(B)**, **(G)**, **(L)**, 20 μm; **(C)**, **(D)**, 10 μm. Bars indicate mean ± SEM.

The authors apologize for this error and state that this does not change the scientific conclusions of the article in any way. The original article has been updated.

