# Supplementary figures and images for: PlexinD1 signaling controls domain-specific dendritic development in newborn neurons in the postnatal olfactory bulb
Source: Front Neurosci. 2023 Jul 13;17:1143130. doi: 10.3389/fnins.2023.1143130 (PMC10393276; doi:10.3389/fnins.2023.1143130)

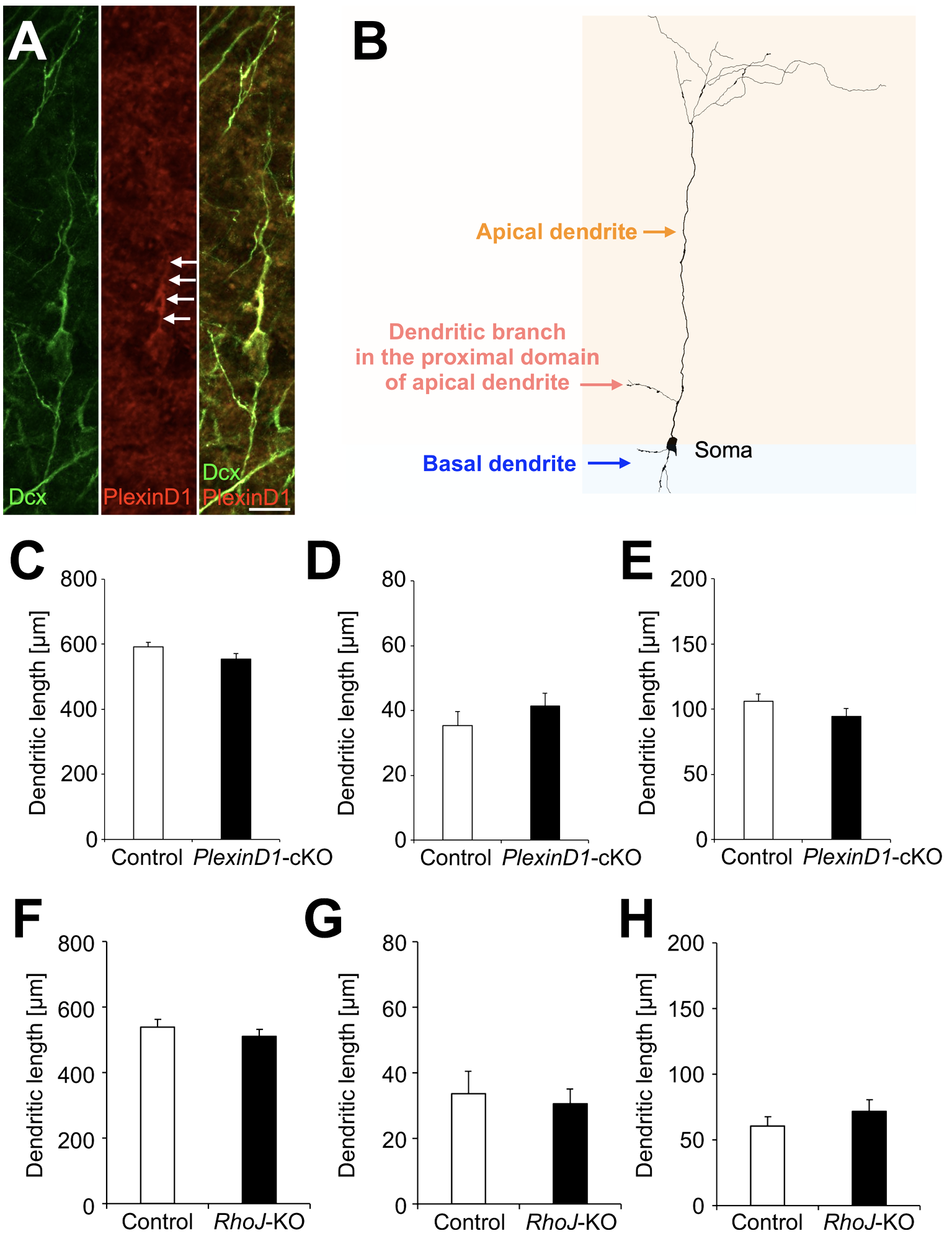

Supplement: SUPPLEMENTARY FIGURE S1 — Dendritic length of newborn granule cells in PlexinD1-cKO and RhoJ-KO mice (A) Representative image of differentiating granule cells stained for Dcx (green) and PlexinD1 (red). Arrows indicate the signal of PlexinD1 protein in the proximal domain of the apical dendrite. (B) Classification of dendrites in newborn granule cells. (C–H) Length of apical dendrites (C, F), dendritic branch in the proximal domain of the apical dendrite (D, G), and basal dendrites (E, H) of granule cells in PlexinD1-cKO (C–E) and RhoJ-KO (F–H) mice. Scale bar, 10 µm. Bars indicate mean ± SEM. [file Image_1.tif]
